# Shared and Disorder-Specific Event-Related Brain Oscillatory Markers of Attentional Dysfunction in ADHD and Bipolar Disorder

**DOI:** 10.1007/s10548-018-0625-z

**Published:** 2018-02-07

**Authors:** Giorgia Michelini, Viryanaga Kitsune, Isabella Vainieri, Georgina M. Hosang, Daniel Brandeis, Philip Asherson, Jonna Kuntsi

**Affiliations:** 1 Institute of Psychiatry, Psychology and Neuroscience, Social, Genetic and Developmental Psychiatry Centre, King’s College London, De Crespigny Park, London, SE5 8AF UK; 2 Centre for Psychiatry, Wolfson Institute of Preventive Medicine, Barts & The London School of Medicine & Dentistry, Queen Mary, University of London, London, UK; 3 Department of Child and Adolescent Psychiatry and Psychotherapy, Central Institute of Mental Health, Medical Faculty Mannheim/Heidelberg University, Mannheim, Germany; 4 Department of Child and Adolescent Psychiatry and Psychotherapy, Psychiatric Hospital, University of Zurich, Zurich, Switzerland; 5 Center for Integrative Human Physiology, University of Zurich, Zurich, Switzerland; 6 Neuroscience Center Zurich, University of Zurich, Zurich, Switzerland

**Keywords:** ADHD, Bipolar disorder, EEG, Brain oscillations, Event-related potentials, Adults

## Abstract

Attention-deficit/hyperactivity disorder (ADHD) and bipolar disorder (BD) often present with overlapping symptoms and cognitive impairments, such as increased fluctuations in attentional performance measured by increased reaction-time variability (RTV). We previously provided initial evidence of shared and distinct event-related potential (ERP) impairments in ADHD and BD in a direct electrophysiological comparison, but no study to date has compared neural mechanisms underlying attentional impairments with finer-grained brain oscillatory markers. Here, we aimed to compare the neural underpinnings of impaired attentional processes in ADHD and BD, by examining event-related brain oscillations during a reaction-time task under slow-unrewarded baseline and fast-incentive conditions. We measured cognitive performance, ERPs and brain-oscillatory modulations of power and phase variability in 20 women with ADHD, 20 women with BD (currently euthymic) and 20 control women. Compared to controls, both ADHD and BD groups showed increased RTV in the baseline condition and increased RTV, theta phase variability and lower contingent negative variation in the fast-incentive condition. Unlike controls, neither clinical group showed an improvement from the slow-unrewarded baseline to the fast-incentive condition in attentional P3 amplitude or alpha power suppression. Most impairments did not differ between the disorders, as only an adjustment in beta suppression between conditions (lower in the ADHD group) distinguished between the clinical groups. These findings suggest shared impairments in women with ADHD and BD in cognitive and neural variability, preparatory activity and inability to adjust attention allocation and activation. These overlapping impairments may represent shared neurobiological mechanisms of attentional dysfunction in ADHD and BD, and potentially underlie common symptoms in both disorders.

**Electronic supplementary material:**

The online version of this article (10.1007/s10548-018-0625-z) contains supplementary material, which is available to authorized users.

## Introduction

The abilities to regulate alertness and sustain attention are essential for efficient information processing and behavior (Posner and Petersen 1990). Such cognitive processes are traditionally measured with reaction time variability (RTV), capturing the consistency and short-term fluctuations in response speed during attentional performance in cognitive tasks (Kuntsi et al. 2013; Ode et al. 2011). Increases in RTV are characteristic of several psychiatric disorders (Kaiser et al. 2008), including attention-deficit/hyperactivity disorder (ADHD) (Kofler et al. 2013; Wood et al. 2010) and bipolar disorder (BD) (Brotman et al. 2009; Moss et al. 2016). ADHD and BD are common psychiatric conditions in adults (Merikangas et al. 2011; Willcutt 2012), which severely impact many aspects of individuals’ lives (Asherson et al. 2014; Skirrow et al. 2012). Although ADHD and BD represent distinct disorders, they present with common symptoms of distractibility and difficulty concentrating, which can lead to uncertainty regarding the boundaries of the two disorders (Asherson et al. 2014; Kitsune et al. 2016). These overlapping symptoms may reflect, at the cognitive level, the common fluctuations in attentional performance and increased RTV displayed by individuals with ADHD and BD (Albaugh et al. 2017; Kuntsi et al. 2014). Increased RTV is also observed in unaffected first-degree relatives of individuals with either disorder, compared to individuals without family risk, representing a candidate marker of genetic/familial risk for both disorders (Adleman et al. 2014; Andreou et al. 2007). Direct comparisons of impairments in attentional performance between ADHD and BD may lead to new insights into the pathways to overlapping symptoms and cognitive dysfunction in both disorders. Yet, cross-disorder comparisons in ADHD and BD are limited to date (Michelini et al. 2016; Rommel et al. 2016; Torralva et al. 2011).

Previous research on RTV in psychiatric disorders has addressed the question of whether dysfunctions in alertness and attentional performance, rather than being stable, could be malleable and sensitive to context changes, such as task manipulations. RTV impairments in children and adolescents with ADHD are maximal in slow and unrewarded conditions, but with the introduction of faster event rate and incentives may improve significantly more than in neurotypical individuals (Andreou et al. 2007; Cheung et al. 2017; Kuntsi et al. 2013; Slusarek et al. 2001; Uebel et al. 2010). It remains unknown, however, whether RTV also improves in adults with ADHD. Initial evidence also indicates potential malleability of RTV in BD, as suggested by one study showing increased RTV in individuals with BD in a continuous performance task (CPT) with low target frequency, but not with high target frequency (Moss et al. 2016). The evidence of malleability in RTV is clinically relevant, as it may point to room for improvement in the observed cognitive impairment, which could be targeted in new interventions for the disorders, aimed at reaching and maintaining an optimal state of alertness (Cheung et al. 2017; Kuntsi and Klein 2012). Understanding whether the same or different mechanisms underlie attentional fluctuations and their potential reduction in individuals with ADHD and BD may thus potentially inform the development of interventions for ADHD and BD. No study to date, however, has compared adults with ADHD and adults with BD on the malleability of attentional fluctuation indexed by RTV.

The investigation of brain responses using the millisecond temporal precision of electroencephalography (EEG) can help elucidate the neural correlates of a suboptimal attentional performance. Most EEG studies on attentional impairments in ADHD or BD samples have employed event-related potentials (ERPs), measuring transient enhancements in brain activity following an event (Luck 2014). ERP studies in adults with ADHD have shown attenuated contingent negative variation (CNV) components over central regions (reflecting atypical response anticipation and preparation) (McLoughlin et al. 2010; Michelini et al. 2016; Valko et al. 2009) and reduced attentional P3 components over parietal regions (reflecting impaired attentional resource allocation) (Cheung et al. 2017, 2016; McLoughlin et al. 2010; Szuromi et al. 2011). Similarly, impairments in P3 and CNV in BD during attentional tasks have also been found (Fridberg et al. 2009; Li et al. 2015; Maekawa et al. 2013). Yet, only a few direct comparisons have examined whether cognitive and ERP indices are affected to a similar extent in ADHD and BD. In a recent investigation using a cued CPT paradigm, we showed that increased RTV and reduced CNV may represent shared attentional impairments in ADHD and BD (Michelini et al. 2016). Using quantitative EEG (QEEG), we further reported that both ADHD and BD groups showed higher spontaneous EEG theta power during rest and a lack of a task-related increase in theta from rest to CPT task compared to controls (Rommel et al. 2016). These results indicate potentially shared impairments in attentional processes in both disorders. Yet, in ERP analyses, the attentional P3 components in response to cue and target stimuli were intact in both groups, consistent with other studies that also failed to report P3 reductions in adults with ADHD (Dhar et al. 2010; Grane et al. 2016; Michelini et al. 2016) or BD (Bestelmeyer 2012; Chun et al. 2013; Michelini et al. 2016). One possible reason for inconsistencies between studies using different attentional paradigms is that the attentional P3, similar to RTV, may reflect a context-dependent and potentially malleable, rather than stable, impairment (Cheung et al. 2017). We recently reported that a reduced parietal P3 in a slow and unrewarded condition in adolescents and young adults with ADHD improved with faster event rate and rewards significantly more than in neurotypical controls (Cheung et al. 2017). In contrast, for CNV, the ADHD group showed reduced amplitude compared to controls only in the fast and rewarded condition. No study has examined the malleability of these ERPs with faster rate and incentives in BD. Further direct comparisons between ADHD and BD are needed to clarify what neurophysiological impairments overlap between the two disorders, and whether ADHD and BD may show similar malleability with a changed context.

Advances in EEG methods called time–frequency analyses, combining the strengths of ERP and QEEG methods, further allow to capture event-related brain oscillatory dynamics, which reflect sub-second modulations of power and phase in response to an event across the full EEG spectrum (Klimesch 1999; Loo et al. 2015; Makeig et al. 2004; Pfurtscheller and Lopes da Silva 1999). Processing and focusing attention on a relevant stimulus have been associated with various event-related brain oscillatory phenomena in the time–frequency domain not captured by ERP or QEEG approaches: (1) an event-related synchronization (ERS) or increase in theta (3–7 Hz) power over fronto-central (Bickel et al. 2012; Lenartowicz et al. 2014; Mazaheri et al. 2014) or parietal (Babiloni et al. 2004; Jacobs et al. 2006) regions, reflecting the initial processing of the stimulus; (2) an event-related desynchronization (ERD) or suppression of power in posterior alpha (8–13 Hz), reflecting attentional selection and cortical activation (Klimesch 2012; Mazaheri and Picton 2005); and (3) an ERD in central beta (14–30 Hz) when a motor response is required (Guntekin et al. 2013; Pfurtscheller 1981). Additionally, indices of consistency of the phase (i.e., the “timing”) of brain oscillations over trials can reveal whether the processing of a stimulus repeated over time reflects stable or variable neural mechanisms (Klimesch 2012; Makeig et al. 2004; Papenberg et al. 2013). Greater alpha and beta ERD and theta phase consistency have further been associated with better task performance (Bickel et al. 2012; Klimesch 2012; McLoughlin et al. 2014). Multiple brain-oscillatory correlates of attentional processes may be affected in ADHD and BD. Individuals with ADHD have been reported to show reductions in event-related phase consistency in the theta band (Groom et al. 2010; McLoughlin et al. 2014), alpha ERD (Lenartowicz et al. 2014; ter Huurne et al. 2013), and beta ERD (Hasler et al. 2016). Emerging evidence also suggests that individuals with BD show attenuations in event-related theta (Atagun et al. 2013; Ethridge et al. 2012) and alpha power (Basar et al. 2012; Ethridge et al. 2012) and increases in beta power (Ozerdema et al. 2013; Tan et al. 2016). These studies in BD, however, applied time–frequency analyses on averaged ERP responses, thus not allowing the characterization of both ERD and ERS dynamics (Bickel et al. 2012). The investigation of fine-grained brain-oscillatory indices underlying attentional processes with time–frequency analyses may allow a deeper investigation into the neural correlates of attentional performance, and help identify distinct or comparable impairments in neural processes between the two disorders (Loo et al. 2015). However, no study to date has compared ADHD and BD on time–frequency indices of brain oscillations, or whether these indices, like RTV, show adjustments under context changes, such as fast and rewarded conditions.

The present study aims to investigate and compare cognitive-performance, ERP and detailed event-related power modulations of theta, alpha and beta oscillations and of phase variability in theta oscillations, previously linked to attentional processes, in adults with ADHD and adults with BD. We used an all-female sample, to match the groups on sex but also because little is known on these processes in females, especially in relation to ADHD (McLoughlin et al. 2010; Saville et al. 2015). Participants completed the same four-choice reaction time task used in our previous studies of ADHD (Andreou et al. 2007; Cheung et al. 2017; Kuntsi et al. 2006), which compares a slow-unrewarded baseline condition with a fast-incentive condition designed to specifically reward reduction of RTV. A further aim is to examine whether differences in adjustments in the investigated cognitive-performance, ERP and brain-oscillatory indices with a faster event rate and incentives emerge between groups, which could inform the development of cognitive/brain training programs for ADHD and BD.

## Methods

### Sample

The sample consisted of 20 women with ADHD, 20 with BD and 20 control women, aged between 20 and 52 years (Table 1). Full information on recruitment and clinical assessment of this sample can be found elsewhere (Kitsune et al. 2016). Briefly, participants with ADHD were recruited from the National Adult ADHD Clinic at the Maudsley Hospital, where any adult female cases meeting inclusion criteria were considered for potential inclusion in the study. Participants with BD were recruited from the Maudsley Psychosis Clinic and a sample that had previously participated in another research study (Hosang et al. 2012). Control participants were recruited from the Mindsearch volunteer database maintained by the Institute of Psychiatry, Psychology and Neuroscience, King’s College London, which comprises several thousand potential participants. Participants were randomly selected from all those meeting recruitment criteria for this study.

**Table 1:** Sample demographics divided by group, with ANOVA test for group differences

	ADHD Mean (SD)	BD Mean (SD)	Ctrl Mean (SD)	F	p
Age	37.4 (7.7)	40.3 (7.7)	36.7 (4.3)	1.63	0.21
IQ	104 (17.9)	108 (12.5)	112 (14.2)	1.37	0.26

Group differences on age and IQ were tested with univariate ANOVAs

*ADHD* attention-deficit/hyperactivity disorder, *BD* bipolar disorder, *Ctrl* control group, *F* ANOVA statistic, *p* p value from the ANOVA

Diagnosis in the clinical groups was confirmed by checking medical records for details of diagnosis and psychiatric history, following DSM-IV criteria. Fifty-seven women with ADHD, 75 women with BD, and 120 control women who matched requirements of age, gender and clinical diagnosis based upon DSM-IV criteria were approached to participate. Among individuals willing to participate (30 with ADHD, 29 with BD and 32 controls), participants were selected for inclusion in the study based on the following exclusion criteria (Kitsune et al. 2016). Exclusion criteria for all groups were drug or alcohol dependency in the last 6 months, autism, epilepsy, neurological disorders, brain injury, past ECT treatment, current involvement in another research trial likely to alter symptom severity, pregnancy or a limited proficiency in English language. Individuals with ADHD and individuals with BD with a reported comorbidity of both ADHD and BD were also excluded. Individuals with BD group who were experiencing a manic episode at the time of the assessment were excluded; only participants who were euthymic at the time of participation were included in the BD group. Control participants, who reported a history of psychiatric disorders or who were taking psychiatric medication, were excluded from the study. Comorbidity in the clinical groups and lack of psychiatric disorders in the control group were further assessed through clinical evaluations when participants underwent the cognitive-EEG assessment for this study (Kitsune et al. 2016). An ADHD diagnosis was excluded in the BD group after conducting the Diagnostic Interview for Adult ADHD (DIVA v. 2.0; (Ramos-Quiroga et al. 2016)). A BD diagnosis was excluded in the ADHD group by checking for a history of past episodes of depression or hypomania/mania and evaluating current mood symptoms using the Altman Self-Rating Mania Scale (Altman et al. 1997) and the Beck Depression Inventory (Beck et al. 1996), and current and lifetime ever symptoms using the Young Mania Rating Scale (Young et al. 1978).

All participants underwent a thorough clinical evaluation with gold-standard diagnostic tools as part of this study; full information on clinical profiles and severity of ADHD or BD in the clinical groups can be found in Kitsune et al. 2016. Participants in the ADHD group had a current combined-type diagnosis or an inattentive-type diagnosis with sufficient symptoms of hyperactivity-impulsivity in childhood to meet a childhood combined-type diagnosis, which reflects the typical adult ADHD clinical population (Asherson et al. 2014). Participants in the BD group had a diagnosis of BD Type I, having experienced at least one manic episode lasting 1 week or more in the past, but were euthymic at the time of the assessments. The ADHD and BD groups did not differ significantly on any of the mood scales for current symptoms, but the ADHD group showed significantly greater levels of ADHD symptoms (e.g., total ADHD symptoms according to the DIVA interview: mean = 13.45, SD = 3.02 in the ADHD group, mean = 4.95, SD = 3.27 in the BD group) (Kitsune et al. 2016).

### Procedure

Participants attended a single 4.5-h research session (including breaks) for cognitive-EEG assessment, IQ assessment and clinical interviews. Participants’ IQs were assessed with the Wechsler Abbreviated Scale of Intelligence Fourth Edition (WASI-IV; (Wechsler 1999)) and did not differ between groups. All participants were asked to refrain from caffeinated drinks and nicotine 2 h before assessments. Participants with ADHD were asked to stop taking any stimulant medication prescribed for their ADHD 48 h prior to the assessment. On the day of the assessments, all ADHD participants who were taking stimulant medication (n = 13) confirmed that they had stopped medication in the preceding 48 h. For ethical reasons, participants were not asked to stop taking mood stabilizers (70% of the BD group), anti-psychotic medication (40% of the BD group) or anti-depressants (7% of the ADHD group and 25% of the BD group) they had been prescribed. Ethical approval for the study was granted by the Camberwell St Giles Research Ethics Committee (approval number 11/LO/0438) and all participants provided informed consent.

### Fast Task

The task for this analysis was a computerized four-choice reaction time task which measures performances under a slow-unrewarded and a fast-incentive condition (Andreou et al. 2007; Kuntsi et al. 2006). In both conditions speed and accuracy were emphasized equally. The baseline (slow-unrewarded) condition followed a standard warned four-choice reaction-time task (Fig. S1, Supplementary material). A warning signal (four empty circles, arranged side by side) first appeared on the screen. At the end of the fore-period lasting 8 s (presentation interval for the warning signal), the circle designated as the target signal for that trial was filled (colored) in. The participant was asked to make a compatible choice by pressing the response key that directly corresponded in position to the location of the target stimulus. Following a response, the stimuli disappeared from the screen and a fixed inter-trial interval of 2.5 s followed. If the participant did not respond within 10 s, the trial terminated. First, a practice session was administered, during which the participant had to respond correctly to five consecutive trials. The baseline condition, consisting of 72 trials, then followed.

To investigate the extent to which a response style characterized by slow and variable speed of responding may be reduced, the task includes a comparison condition that uses a fast event rate (fore-period of 1 s) and incentives (Fig. S1, Supplementary material). This condition started immediately after the baseline condition and consisted of 80 trials, with a fixed inter-trial interval of 2.5 s following the response. The participants were told to respond as quickly as possible to each target, in order to win smiley faces and earn real prizes at the end. Participants won a smiley face for responding faster than their own mean reaction time (MRT) during the baseline (first) condition consecutively for three trials. The baseline MRT was calculated here based on the middle 94% of responses (the exclusion of the top and bottom 3% of responses is only used when calculating a baseline MRT for the set-up of the fast-incentive condition, and is not used for analyses), therefore excluding extremely fast and extremely slow responses. The smiley faces appeared below the circles in the middle of the screen and were updated continuously. The fast-incentive condition was always administered after the baseline condition and, as such, did not involve a similar learning phase. Participants earned £5 in cash after the task battery. RTV for correct responses in each condition was measured to assess task performance.

### EEG Recording and Pre-processing

The EEG was recorded from a 62-channel DC-coupled recording system (extended 10–20 montage) (Brain Products, Gilching, Germany), using a 500 Hz sampling-rate, impedances under 10 kΩ, and FCz as the recording reference. The electro-oculograms (EOGs) were recorded from electrodes above and below the left eye and at the outer canthi. EEG recordings were pre-processed and analyzed using the EEGLAB toolbox (Delorme and Makeig 2004) in Matlab (MathWorks, Natick, MA, USA). Researchers were blind to group status during EEG pre-processing and analysis. Raw EEG recording were down-sampled to 256 Hz, re-referenced to the average of all electrodes (turning FCz into an active channel), and digitally filtered using a 0.25 Hz (− 6 dB cut-off) high-pass filter and a 35 Hz (− 6 dB cut-off) low-pass filter. Independent component analysis (ICA) (Jung et al. 2000) was used to identify and remove ocular (blink-related and vertical and horizontal eye movements) and muscular artefacts. Visual inspection was carried out for all trials to manually remove further artefacts. Channels showing technical problems or excessive electrical noise were removed and replaced with topographic spline interpolation after ICA, to estimate a virtual EEG activity based on artefact-free activity from other channels.

### ERP and Time–Frequency Analyses

**Fig. 1:**
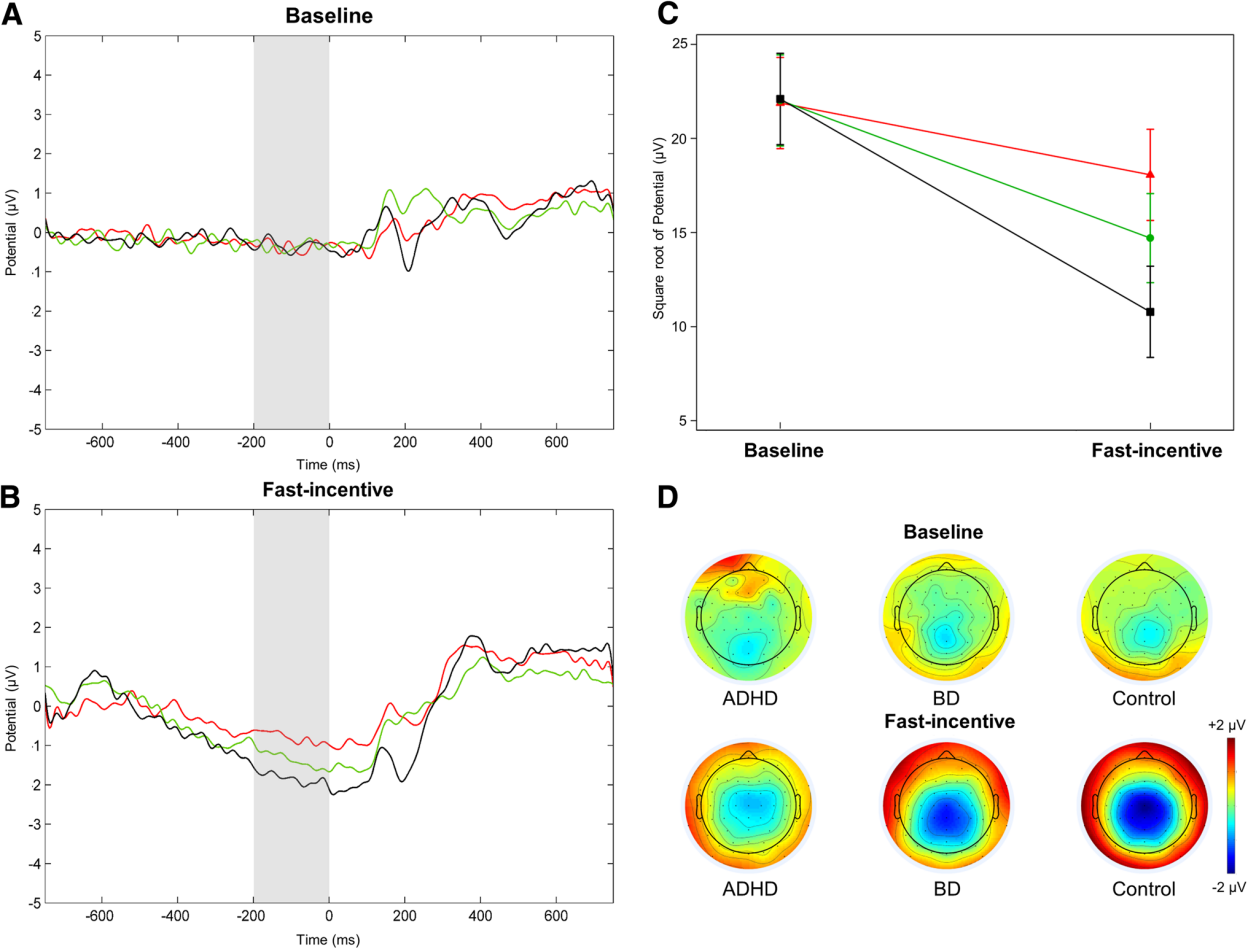
Contingent negative variation (CNV) amplitude measured at Cz in the − 200–0 ms window in the ADHD (in red), BD (in green) and control (in black) groups across the baseline and fast-incentive conditions of the Fast task. **a** Grand average in the baseline condition; **b** grand average in the fast-incentive condition; **c** condition effects by group; **d** topographic maps by group at each condition

Only participants with at least 20 artefact-free EEG segments in each condition were included in ERP/EEG analyses. All ERP/EEG analyses were performed using EEGLAB functions (Delorme and Makeig 2004) and Matlab custom scripts. ERP analyses were restricted to ERP components relevant for attentional processes previously found atypical in studies in ADHD or BD (Cheung et al. 2017; Michelini et al. 2016; Li et al. 2015). ERP peaks were identified within the selected electrodes and latency windows for which effects were expected to be maximal, based on our previous ERP analyses of this task (Cheung et al. 2017; James et al. 2017) and verified against the topographic maps and the grand averages (Fig. 1, Fig. S2, Supplementary material). Following our previous work (Cheung et al. 2017), P3 amplitudes were analyzed at Pz between 300 and 550 ms (Fig. S2, Supplementary material) following the target as the area amplitude measure (µV·ms), to reduce bias due to the varying noise levels induced by the different task conditions (Luck 2014). All trials were baseline-corrected by subtracting the mean activity (200 ms before target onset) from the P3 ERPs. The mean amplitudes of this pre-target period between − 200 and 0 ms were also analyzed separately as a CNV measure at Cz (Fig. 1) with technical zero-baseline approach (which measures the absolute state rather than the amount of neural change introduced by the event) following previous CNV work (Albrecht et al. 2013; Banaschewski et al. 2003; Cheung et al. 2017). This short CNV interval, characterized by a typical CNV topography in the fast-incentive condition with its 1000 ms warning-target interval, was chosen as it captures the late CNV component unconfounded by the processing of warning stimuli. Although no typical CNV emerged in the slower baseline condition, CNV amplitude at Cz in the same corresponding time window was used to examine within-subject change in preparatory activity across conditions.

**Fig. 2:**
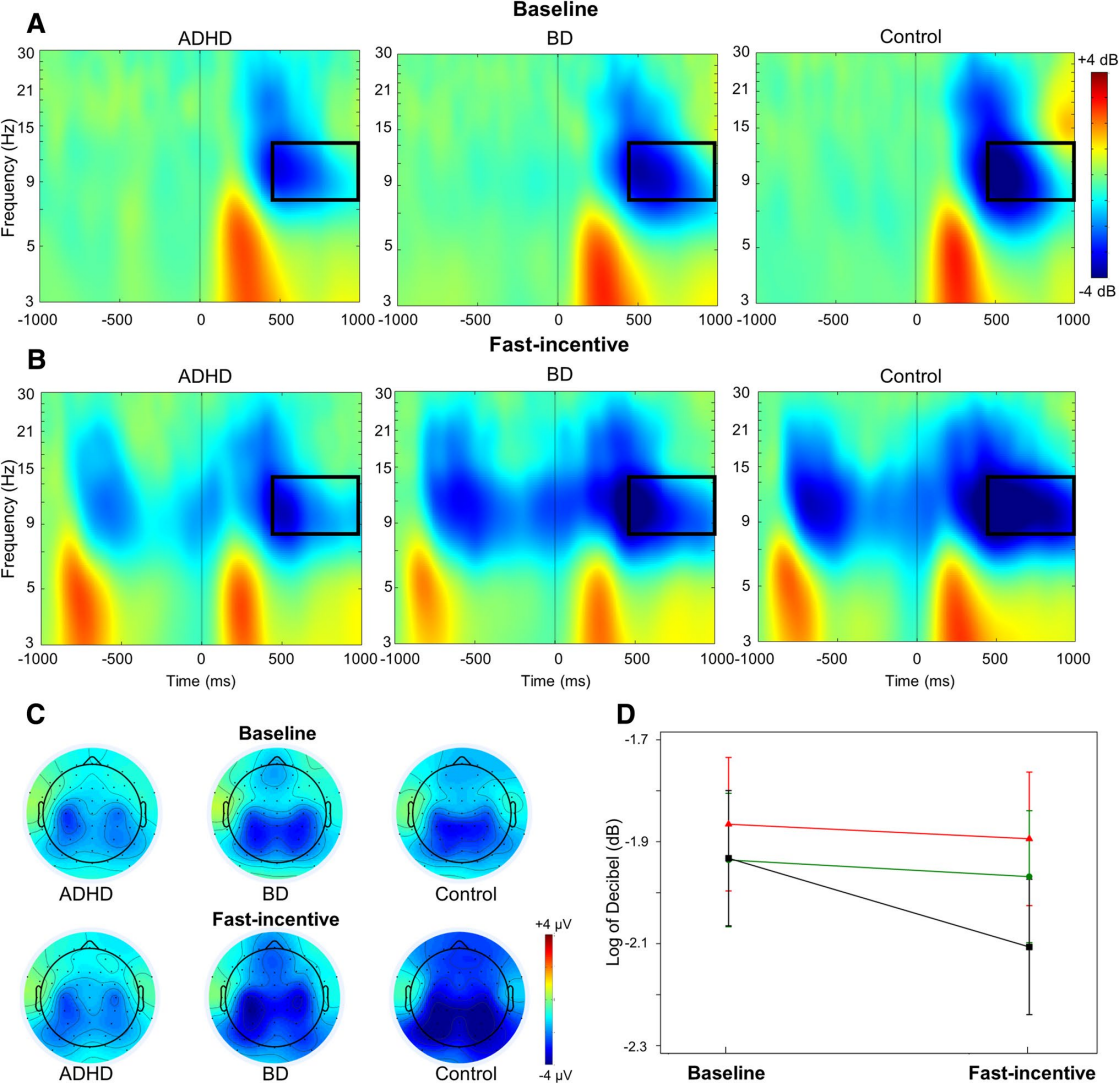
Alpha event-related spectral perturbation (ERSP) at parieto-occipital regions in the ADHD, BD and control groups in the baseline and fast-incentive condition of the Fast task. **a** ERSP in the baseline conditions; **b** ERSP in the fast-incentive condition; **c** topographic maps by group in the 500–1000 ms window at each condition; **d** condition effects in the 500–1000 ms window by group (ADHD group in red, BD group in green, control group in black)

**Fig. 3:**
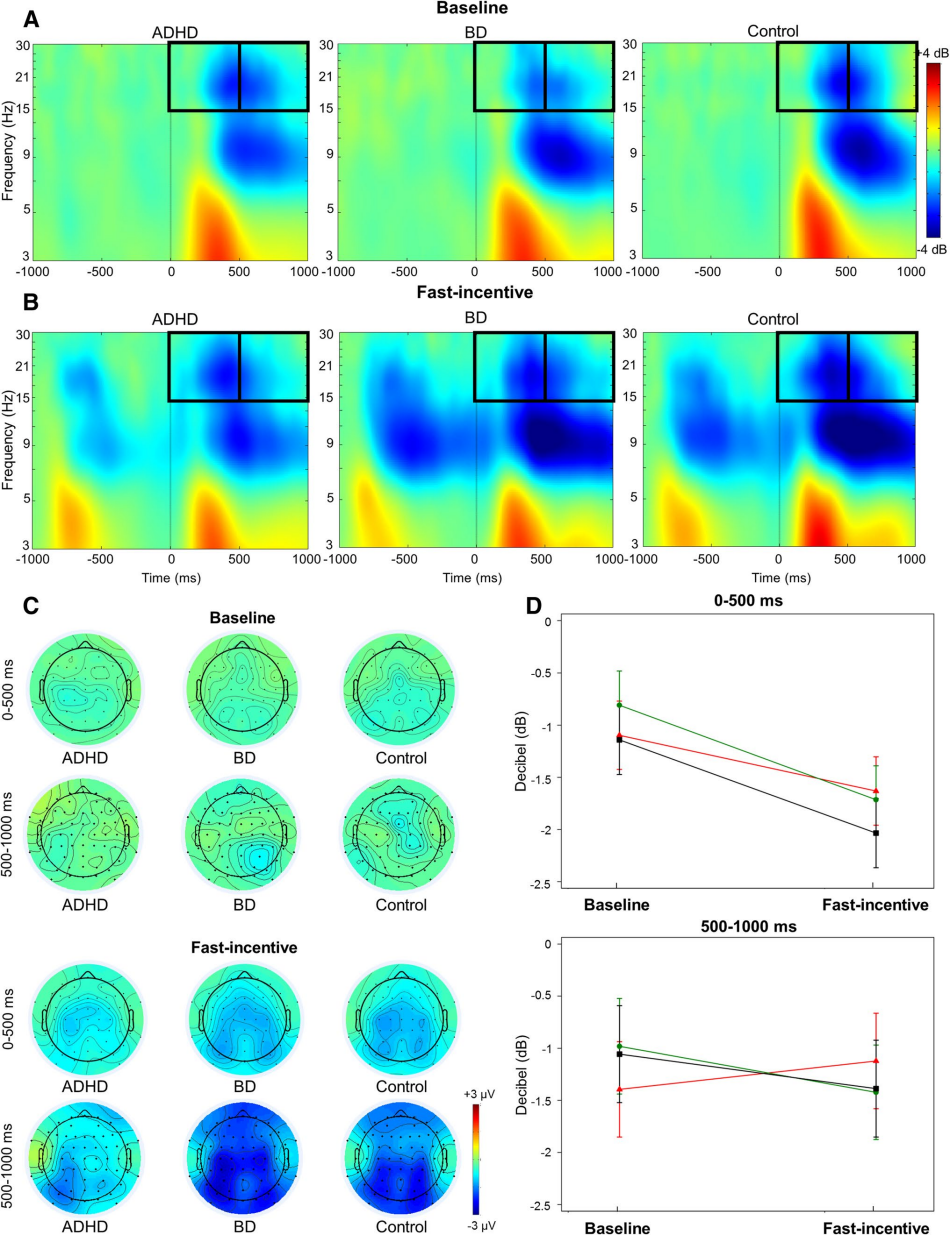
Beta event-related spectral perturbation (ERSP) at central regions in the ADHD, BD and control groups in the baseline and fast-incentive conditions of the Fast task. **a** ERSP in the baseline condition; **b** ERSP in the fast-incentive condition; **c** topographic maps by group in the 0-500 ms and 500–1000 ms windows at each condition; **d** condition effects at each time window by group (ADHD group in red, BD group in green, control group in black)

**Fig. 4:**
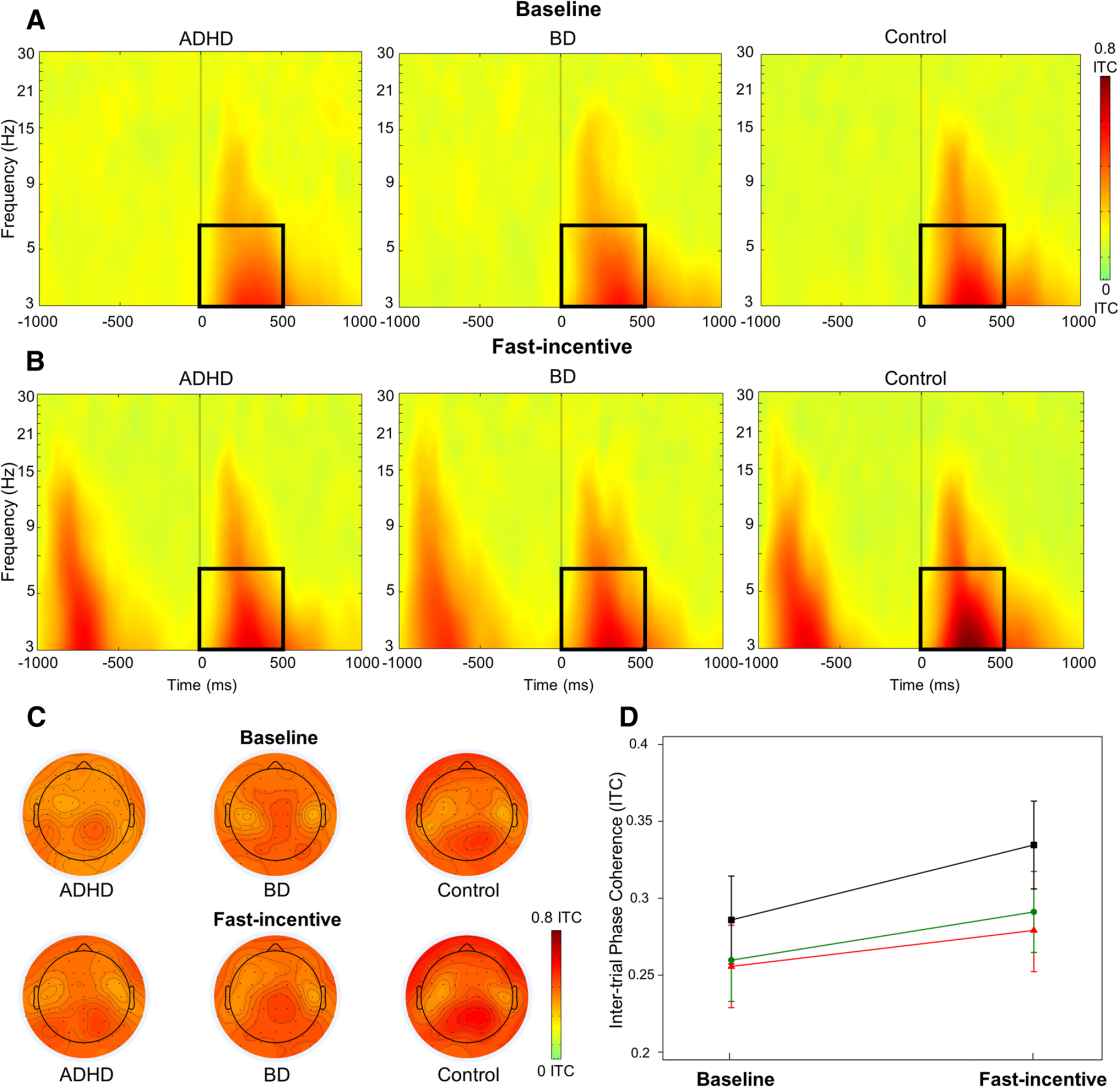
Theta inter-trials phase coherence (ITC) at parietal regions in the ADHD, BD and control groups across the baseline and fast-incentive conditions of the Fast task. **a** ITC in the baseline condition; **b** ITC in the fast-incentive condition; **c** topographic maps by group in the 0-500 ms window at each condition; **d** condition effects in the 500–1000 ms window by group (ADHD group in red, BD group in green, control group in black)

Time–frequency analyses examined the target-related modulations of power and phase consistency of brain oscillations previously implicated in attentional processes and with initial evidence of atypical profiles in ADHD or BD samples based on previous literature (Groom et al. 2010; Hasler et al. 2016; Ethridge et al. 2012). Modulations of power were quantified with the event-related spectral perturbation (ERSP) index (Delorme and Makeig 2004). ERSP values were computed in a 4000 ms window (from − 2000 to 2000 ms) centered around target onset by applying a Morlet wavelet decomposition of frequencies between 3 and 30 Hz, with linearly increasing number of cycles (frequency step of 0.80 Hz) from 2 cycles for the lowest frequency (3 Hz) to 24.60 cycles for the highest frequency (30 Hz). Each ERSP trial was normalized with respect to the mean log-power spectrum from the pre-stimulus period, from − 2000 to − 1000 ms, corresponding to the 1000 ms preceding the warning onset in the fast-incentive condition; the same comparable window was used in the baseline condition as the long fore-period before targets did not produce a modulation of power before stimulus onset in the baseline condition (see Supplementary material for further explanation). Averaging all ERSPs across trials produced a time–frequency representation in decibel (dB) units of increases (ERS, in red) and decrease (ERD, in blue) in the spectral power at a given frequency and latency with respects to pre-stimulus activity (Figs. 2, 3), from which frequency-specific ERSPs can be extracted. Phase consistency was calculated with the inter-trial phase coherence (ITC) index, measuring the degree to which the phase of the evoked response (derived from the same Morlet wavelet used for the ERSP index) at a given latency and frequency is consistent across trials (Delorme and Makeig 2004; Makeig et al. 2004; Tallon-Baudry et al. 1996). ITC values are independent of power, and range from 0 (reflecting absence of phase consistency and highest phase variability across trials) to 1 (indicating perfect phase consistency and lowest phase variability) (Fig. 4).

Target-related ERSP in the theta (3–7 Hz), alpha (8–13 Hz) and beta (14–30 Hz) bands were extracted in the 1000 ms window capturing the broad target-related modulation of power, divided into two consecutive windows for earlier (0–500 ms) and later (500–1000 ms) processing (Figs. 2, 3, Fig. S3, Supplementary material). ITC was measured at target onset in the first window (0–500 ms), where greater phase consistency in response to the event was observed (Fig. 4), as expected (Groom et al. 2010). The ITC analysis was restricted to the theta band, consistent with previous studies reporting a role of this frequency band in neural consistency (Groom et al. 2010; McLoughlin et al. 2014; Papenberg et al. 2013). ERSP and ITC were measured at scalp locations where they were maximal (Figs. 2, 3, 4, Fig. S3, Supplementary material), in line with previous studies on similar attentional processes: theta over parietal regions (average of electrodes: CPz, CP1–CP6, Pz, P3–P4) (DeLosAngeles et al. 2016; Jacobs et al. 2006); alpha over parieto-occipital regions (average of electrodes: Pz, P3–P4, P7–P8, POz, PO3–PO4, PO7–PO8) (Bickel et al. 2012; Mazaheri and Picton 2005); beta over central regions (average of electrodes: Cz, C1–C4, CPz, CP1–CP4) (Bickel et al. 2012; Mazaheri and Picton 2005).

### Statistical Analyses

All measures were investigated using random intercept linear models (i.e., multilevel regression models). Main effects of group (ADHD vs BD vs control), condition (baseline vs fast-incentive) and group-by-condition interactions were examined. Significant (p < 0.05) and trend-level (p < 0.10) effects were followed up with post-hoc analyses testing for (1) between-group differences in baseline and fast-incentive conditions separately, and (2) within- and between-group effects of change between conditions with difference scores. Since ERSP indices were measures at two time windows (0–500, 500–1000 ms), we tested for three-way group-by-condition-by-time interactions for these measures, followed by additional post-hoc tests examining group differences in each time window. Since groups did not differ on IQ or age (Table 1), these variables were not controlled for in analyses. Measures that showed skewed distributions were transformed to normal with square root (CNV, P3) and with logarithm using the “lnskew0” Stata command (MRT, RTV, beta ERSP). In order to inform future larger-scale studies, multiple-testing corrections were deemed not appropriate to limit the chance of introducing type-two errors (i.e. false negative results). Variables for analysis were restricted to measures that were expected to be sensitive to impairments in ADHD or BD to limit the number of hypotheses tested. For between-group comparisons, we report both p-values and Cohen’s d effect sizes (with 95% confidence intervals), calculated using the difference in the means divided by the pooled standard deviation, where d ≥ 0.20 constitutes a small effect, d ≥ 0.50 a medium effect and d ≥ 0.80 a large effect (Cohen 1988). All statistical analyses were run in Stata 14 (Stata Corp, College Station, TX, USA). Data on the fast-incentive condition were missing for one participant with ADHD due to technical issues during the testing session. Two control participants had outlier RTV (> 5 SD) in the baseline condition, indicating that they did not follow task instructions, and were excluded from all analyses. As at least 20 artefact-free EEG segments are needed to obtain reliable ERP/EEG indices (McLoughlin et al. 2009), one participant with ADHD and one with BD were excluded from ERP/EEG analyses on the baseline condition, and one control from ERP/EEG analyses on both conditions. Due to the longer fore-period in the baseline condition, the two conditions were matched on the number of trials, but not on length. To control for this, we run the analyses of RTV performance first on the full baseline condition, and separately on a length-matched segment of the baseline (Andreou et al. 2007) (Supplementary material). Condition length was not controlled for in the ERP/EEG analyses, as data from the full baseline condition was required to obtain sufficient (> 20) trials for averaging.

## Results

### RTV

Significant group (p = 0.01) and condition (p < 0.001) effects, but no group-by-condition interaction (p = 0.92), emerged for RTV. Post-hoc tests of group effects showed that the ADHD and the BD groups had significantly increased RTV compared to controls in both conditions, but did not differ significantly from one another (Table 2). Post-hoc analyses of condition effects showed that all three groups had a significant within-group decrease in RTV from the baseline to the fast-incentive condition, with no significant differences between groups in the degree of change between conditions (Table 3). Comparable results were obtained using the length-matched segment of the baseline condition (Supplementary material).

**Table 2:** Group comparison on cognitive and EEG measures in the baseline and fast-incentive condition

	Baseline condition						Fast-incentive condition					
	ADHD vs BD		ADHD vs Ctrl		BD vs Ctrl		ADHD vs BD		ADHD vs Ctrl		BD vs Ctrl	
	d (95% CI)	p	d (95% CI)	p	d (95% CI)	p	d (95% CI)	P	d (95% CI)	p	d (95% CI)	p
RTV	0.19 (− 0.43; 0.81)	0.544	**0.82 (0.14; 1.48)**	0.016*	*0.69* (*0.03; 1.34*)	0.040*	0.20 (− 0.43; 0.82)	0.541	*0.75* (*0.08; 1.40*)	0.027*	*0.66* (*0.00; 1.31*)	0.050*
CNV	0.02 (− 0.62; 0.65)	0.937	0.08 (− 0.58; 0.73)	0.821	0.05 (− 0.60; 0.71)	0.876	*0.56* (− *0.08; 1.20*)	0.089^†^	**1.41 (0.66; 2.14)**	< 0.001**	*0.69* (*0.02; 1.35*)	0.044*
P3	0.02 (− 0.62; 0.65)	0.954	0. 11 (− 0.54; 0.77)	0.736	0.11 (− 0.55; 0.76)	0.751	0.13 (− 0.50; 0.76)	0.686	0.44 (− 0.22; 1.10)	0.193	*0.56* (− *0.10; 1.21*)	0.099
Theta ERSP (0–500 ms, CP)	0.08 (− 0.56; 0.71)	0.818	0.20 (− 0.46; 0.85)	0.561	0.11 (− 0.54; 0.77)	0.735	0.11 (− 0.52; 0.74)	0.729	0.23 (− 0.43; 0.88)	0.497	0.31 (− 0.35; 0.96)	0.353
Theta ERSP (0–500 ms, FC)	0.31 (− 0.33; 0.95)	0.341	0.06 (− 0.60; 0.71)	0.859	0.17 (− 0.49; 0.82)	0.614	0.19 (− 0.44; 0.81)	0.565	0.25 (− 0.41; 0.90)	0.462	0.41 (− 0.25; 1.06)	0.221
Alpha ERSP (0–500 ms)	0.30 (− 0.35; 0.93)	0.368	0.39 (− 0.28; 1.04)	0.389	0.13 (− 0.62; 0.65)	0.967	*0.50* (− *0.14; 1.13*)	0.129	0.48 (− 0.19; 1.14)	0.160	0.04 (− 0.61; 0.68)	0.908
Alpha ERSP (500–1000 ms)	0.29 (− 0.35; 0.92)	0.382	0.31 (− 0.35; 0.96)	0.363	0.04 (− 0.61; 0.70)	0.896	0.27 (− 0.36; 0.90)	0.399	*0.78* (*0.09; 1.45*)	0.026*	0.44 (− 0.21; 1.09)	0.191
Beta ERSP (0–500 ms)	0.38 (− 0.27; 1.02)	0.251	0.17 (− 0.49; 0.82)	0.613	*0.52* (− *0.15; 1.18*)	0.129	0.10 (− 0.53; 0.72)	0.764	*0.56* (− *0.12; 1.22*)	0.105	0.38 (− 0.27; 1.04)	0.248
Beta ERSP (500–1000 ms)	0.35 (− 0.31; 0.97)	0.309	0.46 (− 0.21; 1.11)	0.179	0.02 (− 0.63; 0.68)	0.943	0.30 (− 0.34; 0.93)	0.362	0.30 (− 0.36; 0.95)	0.378	0.04 (− 0.61; 0.69)	0.905
Theta ITC	0.09 (− 0.54; 0.72)	0.787	0.47 (− 0.20; 1.13)	0.168	0.43 (− 0.23; 1.10)	0.199	0.17 (− 0.46; 0.80)	0.600	**0.83 (0.14; 1.51)**	0.018*	*0.72* (*0.05; 1.38*)	0.036*

*95% CI* 95% confidence intervals around d estimates, *ADHD* attention-deficit/hyperactivity disorder, *BD* bipolar disorder, *CNV* contingent negative variation, *CP* centro-parietal region, *Ctrl* control group, *d* Cohen’s d, *ERSP* event-related spectral perturbation, *FC* fronto-central region, *ITC* inter-trial phase coherence, *MRT* mean reaction time, *p* p value from mixed models, *RTV* reaction time variability

**p < 0.01, *p < 0.05, ^†^p < 0.10. Bold = large effect size (d ≥ 0.80); Italics = medium effects size (d ≥ 0.50)

**Table 3:** Comparison of condition effects within group and between groups

	Within-group differences			Between-group differences					
	ADHD	BD	Ctrl	ADHD vs BD		ADHD vs Ctrl		BD vs Ctrl	
	p	p	p	d (95% CI)	p	d (95% CI)	p	d (95% CI)	p
RTV	< 0.001**	< 0.001**	< 0.001**	0.01 (− 0.61, 0.93)	0.982	0.22 (− 0.42; 0.86)	0.507	0.30 (− 0.35; 0.94)	0.366
CNV	0.019*	< 0.001**	< 0.001**	*0.59* (− *0.08; 1.24*)	0.083†	**1.17 (0.44; 1.87)**	0.002**	*0.59* (− *0.09; 1.25*)	0.088†
P3	0.723	0.331	0.026*	0.08 (− 0.56; 0.72)	0.814	0.49 (− 0.19; 1.16)	0.159	*0.68* (*0.12; 1.32*)	0.048*
Theta ERSP (0–500 ms, CP)	0.039*	0.003**	0.085†	0.19 (− 0.45; 0.83)	0.567	0.03 (− 0.63; 0.69)	0.930	0.21 (− 0.45; 0.86)	0.543
Theta ERSP (0–500 ms, FC)	0.004*	< 0.001**	0.056†	0.40 (− 0.25; 1.05)	0.231	0.17 (− 0.49; 0.84)	0.612	0.55 (− 0.11; 1.22)	0.106
Alpha ERSP (0–500 ms)	0.001**	< 0.001**	< 0.001**	0.44 (− 0.21; 1.09)	0.188	0.44 (− 0.23; 1.10)	0.202	0.05 (− 0.60; 0.70)	0.879
Alpha ERSP (500–1000 ms)	0.568	0.510	< 0.001**	0.13 (− 0.52; 0.77)	0.701	*0.71* (*0.02; 1.39*)	0.045*	*0.52* (− *0.15; 1.18*)	0.132
Beta ERSP (0–500 ms)	< 0.001**	< 0.001**	< 0.001**	*0.69* (*0.02; 1.35*)	0.044*	*0.72* (*0.03; 1.40*)	0.040*	0.05 (− 0.61; 0.70)	0.885
Beta ERSP (500–1000 ms)	0.104	0.007**	0.054†	**1.05 (0.35; 1.73)**	0.003**	**0.87 (0.17; 1.56)**	0.014*	0.14 (− 0.52; 0.79)	0.683
Theta ITC	0.083†	0.018*	< 0.001**	0.16 (− 0.49; 0.80)	0.634	0.43 (− 0.25; 1.09)	0.216	0.30 (− 0.36; 0.95)	0.379

*95% CI* 95% confidence intervals around d estimates, *ADHD* attention-deficit/hyperactivity disorder, *BD* bipolar disorder, *CNV* contingent negative variation, *CP* centro-parietal region, *Ctrl* control group, *d* Cohen’s d, *ERSP* event-related spectral perturbation, *FC* fronto-central region, *ITC* inter-trial phase coherence, *MRT* mean reaction time, *p* p value from mixed models, *RTV* reaction time variability

**p < 0.01, *p < 0.05, †p < 0.10. Bold = large effect size (d ≥ 0.80); Italics = medium effects size (d ≥ 0.50).

### ERPs

#### CNV

Significant main effects of group (p = 0.03) and condition (p < 0.001), and a significant group-by-condition interaction (p < 0.01), emerged for the CNV. Post-hoc tests showed no group differences in the baseline condition (Table 2). In the fast-incentive condition, the CNV was significantly reduced in the ADHD compared to the control group (Fig. 1). The BD group showed a trend-level effect for greater CNV compared to the ADHD group, and a significantly reduced CNV compared to controls (Table 2). All three groups had a significant within-group decrease from the baseline to the fast-incentive condition (Table 3; Fig. 1). The degree of change in CNV between conditions in the ADHD group was significantly lower compared to the control group, and at trend level compared to the BD group. The BD group also showed a trend-level reduction in CNV compared to the control group in the degree of change between conditions.

#### P3

A trend-level group-by-condition interaction (p = 0.06), but no main effects of group (p = 0.84) or condition (p = 0.56), emerged for the P3. Post-hoc tests did not show significant group differences in the baseline or in the fast-incentive condition (Table 2, Fig. S2, Supplementary material). A significant within-group change from the baseline to the fast-incentive condition in stimulus-locked P3 emerged in controls, but not in participants with ADHD or BD (Table 3). The degree of change between conditions was significantly lower in the BD compared to the control group. The ADHD group did not differ significantly from the other two groups in the degree of change between conditions.

### Event-Related Power (ERSP)

#### Theta

No effects of group (p = 0.96), condition (p = 0.11) or group-by-condition-by-time interaction (p = 0.94) emerged for theta ERSP. After removing the three-way interaction, there were no significant group or group-by-condition interaction effects on this measure (p > 0.61), and a significant main effect of condition emerged in the 0–500 ms window (p < 0.001) but not in the 500–1000 ms window (p = 0.41). In the 0–500 ms window, a significant within-group decrease from the baseline to the fast-incentive condition emerged in theta ERSP for the ADHD and BD groups, and at trend level for the control group (Table 3), but there were no group differences in the degree of change between conditions (Table 3). An additional analysis examined the event-related theta ERSP that was evident also at fronto-central regions (Fig. S3, Supplementary material), yielding the same results as found for parietal theta power (Tables 2, 3).

#### Alpha

A main effect of condition (p < 0.001), but no effects of group (p = 0.25) or group-by-condition-by-time interaction (p = 0.23), emerged for alpha ERSP. After removing the three-way interaction, there was a significant effect of condition (p < 0.001), but no significant group (p = 0.30) or group-by-condition interaction effects in the 0–500 ms time window for this measure (p = 0.48). All three groups showed a significant within-group decrease in alpha ERSP (i.e., increase in alpha suppression) in the change from the baseline to the fast-incentive condition (Table 3), but there were no group differences in the degree of change between conditions. In the 500–1000 ms window, a main effect of condition (p = 0.01), a trend-level group-by-condition interaction (p = 0.08), but no main effect of group (p = 0.23), emerged for alpha ERSP. Post-hoc tests showed no differences between groups in the baseline condition (Table 2). In the fast-incentive condition, the ADHD group showed a significantly decreased alpha ERSP (i.e., lower alpha suppression) compared to controls (Fig. 2). The BD group did not differ from the other groups. A significant within-group decrease from the baseline to the fast-incentive condition in alpha ERSP (i.e., increase in alpha suppression) emerged for the control group, but not for the ADHD or BD groups (Table 3). The ADHD group showed a significantly lower degree of change between conditions than the control group in this measure, while the BD group did not differ from the other groups (Table 3).

#### Beta

A significant main effect of condition (p < 0.001), but no significant effect of group (p = 0.75) or group-by-condition-by-time interaction (p = 0.61), emerged for beta ERSP. After removing the three-way interaction, there was no significant group effects in either time window (p > 0.25), but there were significant condition (p < 0.001) and trend-level group-by-condition interaction (p = 0.06) effects in the in the 0–500 ms window, and significant group-by-condition interaction (p = 0.01) and trend-level condition (p = 0.08) effects in the 500–1000 ms window. A significant within-group decrease in beta ERSP (i.e., increase in beta suppression) from the baseline to the fast-incentive condition emerged for all groups in the 0–500 ms window, but only for control and BD groups in the 500–1000 ms window (Table 3; Fig. 3). The ADHD group differed from the BD and control groups in the degree of change in beta ERSP between conditions in both time windows, while the BD and control groups did not differ from one another (Table 3).

### Theta Phase Consistency (ITC)

A main effect of group (p = 0.03) and condition (p < 0.001), but no group-by-condition interaction (p = 0.41), emerged for theta ITC in the 0–500 ms window. Post-hoc tests showed no differences between groups in the baseline condition (Table 2). In the fast-incentive condition, theta ITC was significantly decreased (i.e., phase was more variable) in the ADHD and BD groups compared to the control group, with no differences between ADHD and BD groups (Fig. 4). A significant within-group increase in theta ITC (i.e., decrease in phase variability) from the baseline to the fast-incentive condition emerged in the control and BD groups, and at trend-level in the ADHD group (Table 3), but no differences between groups emerged in the degree of change between conditions. Further analyses compared groups prior to target onset, and found no differences in theta ITC before target appearance (Supplementary material).

## Discussion

In this comparison between ADHD and BD on cognitive, ERP and brain-oscillatory markers of attentional processes, women with ADHD and women with BD showed overlapping impairments in fluctuations in attentional performance (RTV), neural variability (theta ITC) and neural response preparation (CNV). Individuals with either disorder further displayed a similar inability to adjust neural attention allocation (P3) and activation (alpha suppression) from a baseline to a fast-paced and rewarded condition, suggesting no adaptation to a changed context in these processes. Additional disorder-specific alterations in alpha and beta suppression were displayed by women with ADHD only, but impairments in most processes were shared between the two disorders. By examining both ERP and fine-grained brain-oscillatory indices of brain activity, these findings reveal novel neural mechanisms of shared attentional dysfunction in ADHD and BD, which potentially underlie some of the common symptoms in both disorders.

At the cognitive level, both ADHD and BD groups showed increased RTV in both task conditions, indicating more frequent fluctuations in response speed and impairments in the ability to sustain attention during the task. Increased RTV in both disorders is consistent with our results with this sample using a cued CPT task (Michelini et al. 2016), and previous studies on ADHD (Cheung et al. 2016; Kofler et al. 2013; Kuntsi et al. 2010) and BD (Bora et al. 2006; Brotman et al. 2009; Moss et al. 2016). We further show novel evidence of intra-individual variability also at the neural level in the phase of theta oscillations in both women with ADHD and women with BD. Low phase variability over trials is thought to reflect an adaptive mechanism to maintain stable neural processing of a stimulus (Makeig et al. 2004; Papenberg et al. 2013). The increased variability in theta oscillations, previously reported in adolescents with ADHD (Groom et al. 2010; McLoughlin et al. 2014), thus points to increased variability in the timing of evoked theta responses to targets over trials in adults with ADHD and BD (Cavanagh et al. 2009; McLoughlin et al. 2014). Although these differences emerged as significant only in the fast-incentive condition, the group-by-condition interaction was not significant, suggesting that there may be subtle differences also in the baseline condition, non-significant in this sample. Further analyses in the pre-stimulus window indicated that, compared to individuals with ADHD or BD, control women displayed greater phase consistency upon target presentation, but lower consistency before targets. As such, with presentations of targets across trials, the controls displayed a consistent alignment and increase in consistency in the phase of theta (called phase resetting) (Lakatos et al. 2009; Palaniyappan et al. 2012) from the low consistency observed in the pre-stimulus window. This mechanism may be lacking in women with either ADHD or BD as indicated by the more frequent fluctuations in this neural mechanism across trials. Overall, our findings of increased variability in cognitive and neural processes in women with ADHD or BD indicate an overlap in the neural underpinnings of impaired attentional fluctuations in both disorders, which may point to common neurobiological dysfunctions.

By further examining pre-stimulus response preparation in ADHD and BD, we found shared preparatory impairments, as indicated by reduced CNV, in both clinical groups in the fast-incentive condition. This finding is consistent with our previous results in this sample using a CPT task (Michelini et al. 2016), and in adolescents and young adults with ADHD using the same task employed in this study (Cheung et al. 2017). Suggestive (trend-level) differences between ADHD and BD in this measure may also indicate more pronounced CNV impairment in ADHD, although this awaits replication in future studies. The pattern for P3 amplitude in response to targets, which was not different from controls in either ADHD or BD groups, indicates that women with either disorder may not be impaired in this ERP of attentional allocation. This result is consistent with our previous study with this sample (Michelini et al. 2016), showing intact P3s following cue and target stimuli, and other previous studies reporting normal attentional P3 amplitudes in adults with ADHD (Barry et al. 2009; McLoughlin et al. 2010) or BD (Bestelmeyer 2012). Yet, this P3 finding does not align with our previous larger-scale investigation using this task in ADHD, where our predominantly-male group of adolescents and young adults with ADHD (mean age: 18 years) showed a reduced P3 in the baseline condition (with a small effect size) compared to controls (Cheung et al. 2017). In the current study, the intact target P3 in ADHD may be due to gender or age, the present study being the first on this task using an all-female and all-adult sample (mean age: 37 years). In addition, the ADHD group had lower IQ than the control group in our previous study, and the ADHD-control difference on the P3 was non-significant when IQ was controlled for (Cheung et al. 2017). The lack of IQ differences between groups in the current sample may have contributed to the lack of group differences in the P3. Taken together, these findings indicate that both ADHD and BD are associated with reduced ERP activity of attentional preparation and anticipation of motor responses.

With faster target presentation and incentives, further shared impairments between ADHD and BD emerged in adjustments between conditions. These task manipulations, originally designed in ADHD studies to reward more consistent response times, produced comparable reductions in RTV in clinical and control groups. At the neural level, women with ADHD, and potentially (at trend-level) with BD, displayed significantly reduced increases in CNV amplitude compared to controls, and no improvements in allocation of attentional resources (P3) (Polich 2007) or attentional selection (alpha suppression) (Klimesch 2012). The novel finding of a reduced ability to increase alpha suppression with task demands in both disorders points to a common inability in individuals with ADHD and BD to regulate brain activity implicated in attentional selection processes (Klimesch 2012; Klimesch et al. 2007). A reduced adjustment in the response preparation CNV in women with ADHD replicates our previous findings in adolescents and young adults with the disorder (Cheung et al. 2017). Yet, in the P3, neither of the clinical groups showed the improvement between conditions displayed by controls. This pattern for the P3 contrasts with our previous findings using this task in a sample of adolescents and young adults with ADHD, where the ADHD group showed improvements between conditions in the P3, which were greater than those observed in the control group, suggesting malleability in this attentional ERP component in ADHD (Cheung et al. 2017). Similarly, these results in ADHD do not align with studies in children, adolescents and young adults indicating greater RTV malleability and improvements in ADHD than in neurotypical samples (Andreou et al. 2007; Cheung et al. 2017; Kuntsi et al. 2013, 2009). A possible explanation for the inconsistencies in P3 and RTV adjustments is the age difference between the samples of current and previous studies: it could be hypothesized that adults with ADHD, compared to younger individuals, may be less sensitive to task manipulations in these processes. Gender effects represent another possible reason for inconsistencies with previous studies based on predominantly-male samples (Andreou et al. 2007; Cheung et al. 2017). Longitudinal studies and replications in larger samples, including individuals of both sexes, are needed to examine potential developmental and gender effects on the malleability of markers of attentional processes in ADHD.

While most impairments were shared between ADHD and BD, we further found impairments specific to ADHD relative to controls, which were not displayed by women with BD. Women with ADHD displayed a dysfunction in attentional selection, as indexed by lower alpha power suppression in response to targets in the fast-incentive condition (Klimesch 2012; Klimesch et al. 2007). These results are consistent with previous studies reporting attenuated event-related alpha suppression in ADHD (Hasler et al. 2016; Lenartowicz et al. 2014; Mazaheri et al. 2014; Missonnier et al. 2013). In addition, in the change from the baseline to the fast-incentive condition, individuals with ADHD were specifically associated with lower adjustments in the suppression of beta power than in individuals with BD and controls, indicating reduced improvements in neural mechanisms associated with response execution (Bickel et al. 2012; Mazaheri et al. 2014). The lack of a difference between women with BD and controls on measures of alpha and beta power suppression has not been reported in previous studies of BD, which measured event-related power extracted from averaged ERP responses and thus could not capture event-related power suppressions. Future investigations using the finer-grained time–frequency methods employed in this study are needed to confirm typical profiles in alpha and beta power suppression measures in individuals with BD. While the ADHD-specific impairment in alpha suppression did not distinguish women with ADHD from women with BD, the reduction in the adjustment in beta power suppression with task demands significantly differentiated the two clinical groups. The latter brain-oscillatory process may thus represent neurobiological dysfunctions specific to ADHD, which may potentially help delineate ADHD from BD in adults.

The following limitations should be considered when interpreting our findings. First, although the groups were matched on gender, age and IQ, there were differences in the prescribed medications that participants were taking. We asked participants with ADHD to stop taking stimulant medication 48 h before assessments, but it was not possible, for ethical reasons, to ask participants to stop mood-stabilizing, anti-psychotic or antidepressant medications. Medication effects are difficult to control for in cross-disorder studies where different groups are prescribed different treatments, resulting in a limited number of participants within medication subgroups. However, previous studies suggest that medication may show positive effects (reducing differences from controls) or no effects on cognitive-EEG measures (Anderer et al. 2002; Degabriele and Lagopoulos 2017; Galletly et al. 2005; Karaaslan et al. 2003). As such, it is unlikely that the significant group differences reported in this study reflect confounding medication effects. Yet, the possibility remains that the lack of differences between clinical groups and the control group on some measures (especially for the BD group, where the majority of individuals were taking medication) may be due to medication effects, which may have attenuated case-control differences. Future studies on samples including non-medicated individuals or a higher number of individuals in each medication sub-group are needed to clarify this issue. Second, while the two task conditions were matched on number of trials, they differed in duration and in length of the fore-period between warning and target stimuli. While we obtained comparable findings in RTV with length-matched segments, ERP/EEG analyses could not be repeated on length-matched segments, as doing so would have produced insufficient number of trials in the baseline condition to obtain reliable ERP/EEG indices. In addition, the different fore-periods and the use of a 0.25 Hz high-pass filter may reduce comparability of preparatory activity between the conditions. Yet, the analysis of the CNV (showing typical topographies at central sites) and the further analyses of EEG activity in the warning-target interval under fast-incentive conditions (Supplementary material) allowed detailed investigation of neural preparatory processes in this latter condition. Future studies could examine stimulus-related and preparatory processes in ADHD and BD using other tasks, as well as examine the influences on slower frequencies on the CNV. Third, although the current study and previous analyses on this sample (Michelini et al. 2016; Rommel et al. 2016) represent the most comprehensive comparisons between ADHD and BD on cognitive, ERP and EEG markers to date, the sample is relatively small. While several significant differences between groups emerged with medium-to-large effects with current sample sizes, larger studies are needed to confirm these results and further investigate subtler impairments in ADHD and BD. Finally, the adult participants in the clinical groups recruited for this study showed higher than expected IQ scores, which did not differ from IQ scores in the control group. Future replication in samples with a wider range of IQs is required in order to generalize these findings to more typical clinical populations.

Taken together, these findings further our understanding of the neural underpinnings of attentional impairments in both disorders, and provide new evidence into the overlap and specificity of impairments in these processes in women with ADHD and BD. The shared markers of attentional dysfunctions may represent biomarkers for both disorders. The shared atypical neural profiles related to attentional processes may underlie similarities in behavioral symptoms (e.g., distractibility) between ADHD and BD, which can lead to difficulty in delineating between ADHD and BD and incorrect treatment decisions. Finally, since ADHD and BD show genetic overlap (Lee et al. 2013; Song et al. 2015; van Hulzen et al. 2016), and increased attentional fluctuations may represent candidate markers of genetic/familial risk for both disorders (Adleman et al. 2014; Andreou et al. 2007), future studies could examine whether shared genetic factors may underlie overlapping attentional dysfunctions in ADHD and BD.

## Electronic supplementary material

Below is the link to the electronic supplementary material.

Supplementary material 1 (DOCX 1705 KB)
