# Supplementary material for: Shared and Disorder-Specific Event-Related Brain Oscillatory Markers of Attentional Dysfunction in ADHD and Bipolar Disorder
Source: Brain Topogr. 2018 Feb 7;31(4):672–89. doi: 10.1007/s10548-018-0625-z (PMC5999167; doi:10.1007/s10548-018-0625-z)
Supplement: Supplementary file 1 — Supplementary material 1 (DOCX 1705 KB) [file 10548_2018_625_MOESM1_ESM.docx]

**SUPPLEMENTARY MATERIAL**

## *Shared and disorder-specific event-related brain oscillatory markers of attentional dysfunction in ADHD and bipolar disorder*

*Giorgia Michelini, M.Sc.; Viryanaga Kitsune, Ph.D.; Isabella Vainieri, M.Sc.; Georgina M. Hosang, Ph.D.; Daniel Brandeis, Ph.D.; Philip Asherson, M.R.C. Psych., Ph.D.; Jonna Kuntsi, Ph.D.*

**Analysis of length-matched cognitive-performance indices**

The two conditions were not matched on task length, but were matched on the number of trials. The fore-period in the baseline condition (8 s) was longer than in the fast-incentive condition (1 s). This difference was specifically designed to probe sustained attention and vigilance in attention-deficit/hyperactivity disorder (ADHD) samples in the baseline condition, and test improvements with shorter fore-periods. To exclude the possibility that differences between conditions may be due to differential task length, we analyzed reaction time variability (RTV) performance on both the full baseline condition (presented in the main text) and separately on the length-matched segment, consistent with our previous studies on this task (Andreou et al. 2007; Cheung et al. 2017; Kuntsi et al. 2013). Data from 30 trials of the baseline condition were used, to provide a match on length of time on task with the fast-incentive condition. The second set of 30 trials are used for this analysis, as this segment of the baseline condition was considered more reliable than the first set of 30 trials, which is likely a reflection of an initial learning phase during the first part of the task (Andreou et al. 2007). Since the fast-incentive condition was always administered after the baseline condition, it did not involve a similar learning phase.

A significant main effect of group (p=0.004) and condition (p<0.001), but no group-by-condition interaction (p=0.69) emerged, with both the ADHD and the BD groups showing increased RTV compared to the control group, but no differences between clinical groups, in the length-matched baseline condition (ADHD vs BD: d=0.44, 95% CIs=-0.19–1.07, p=0.17; ADHD vs Control: d=1.04, 95%, CIs=0.35–1.71, p=0.003; BD vs Control: d=0.61, 95% CIs=-0.04–1.26, p=0.067). All three groups showed significant within-group differences between conditions (all p<0.003), but no group differences in the degree of change between conditions (ADHD vs BD: d=0.22, 95% CIs=-0.41–0.84, p=0.50; ADHD vs Control: d=0.29, 95% CIs=-0.35–0.62, p=0.37; BD vs Control: d=0.17, 95% CIs=-0.47–0.81, p=0.595).

These additional analyses show that comparable results to those in the full baseline were obtained for RTV using the length-matched segment of the baseline condition (Andreou et al. 2007; Cheung et al. 2017).

**Further details on the analysis of event-related perturbation (ERSP) indices**

In time-frequency analyses, the modulations of EEG frequency components in response to a stimulus are normalized with respect to spectral power in a pre-defined pre-stimulus period. Specifically, the post-stimulus power at each time-frequency point is divided by the mean spectral power in the pre-stimulus period (typically reflecting spontaneous EEG) at the same frequency (Grandchamp and Delorme 2011; Herrmann et al. 2014). The normalized post-stimulus signal is scaled in decibel (dB), a logarithmic unit that represents the ratio of two signals. When comparing the ERSPs in two conditions, it is necessary to match the pre-stimulus period used to normalize the post-stimulus ERSPs (Herrmann et al. 2014). In the present study, to compare the ERSPs in the baseline and fast-incentive conditions, we matched the timing of the pre-stimulus window across the two conditions (-2 to -1 s) with respect to the appearance of the target stimulus (Figure S1). This window represents the -1 to 0 s period before the warning stimulus appearing 1 s before the target in the fast-incentive condition, reflecting a window of spontaneous EEG activity before warning and target onsets. The same corresponding -2 to -1 s window in the baseline condition similarly represents a period of spontaneous EEG activity during the long fore-period between the appearance of the warning and of the target. This window in the baseline condition was chosen instead of the corresponding pre-warning window in the baseline condition (-9 to -8 ms) because segmenting the data around the target from the pre-warning window in the baseline condition to the post-target period (-9 to 1 s after the target) would have produced too few of such long segments for analyses in the baseline condition.

**Analysis of pre-stimulus theta inter-trial phase coherence (ITC) in the fast-incentive condition**

Since a difference between groups emerged in target-related theta ITC in the fast-incentive condition, we carried out an additional analysis to examine whether these differences could be attributed to differences in the phase of theta prior to target onset. Theta ITC data were calculated as described in the main text, and examined in the -500-0 ms window before appearance of target stimuli. We investigated pre-target theta ITC and post-target theta ITC (measured as described in the main text) in a repeated measures mixed model, testing for main effects of group (ADHD vs BD vs control), time window (pre-target vs post-target) and group-by-window interactions.

A significant effect of time window (p<0.001) and group-by-window interaction (p<0.001), but not main effect of group (p=0.38), emerged for theta ITC. Post-hoc tests showed that groups did not differ in the pre-target time window (overall group effect: p=0.17), but the control group had significantly greater ITC than the ADHD and BD groups in the post-target time window, as reported in the main text. All three groups showed a significant increase in theta ITC from the pre-target to the post-target time window (all p<0.001), but the control group showed a greater degree of change between time windows than the ADHD (d=1.05, 95% CIs=0.34–1.74, p=0.003) and BD (d=1.10, 95% CIs=0.40–1.79, p=0.002) groups. The ADHD and BD groups did not differ in the change between time windows (d=0.08, 95% CIs=-0.55–0.70, p=0.81).

These further analyses indicate that greater phase consistency following target stimuli in the control group, compared to the clinical groups, cannot be attributed to differences in the pre-target window: in the pre-target window, all groups show lower theta ITC values than in the post-target window, as expected (Makeig et al. 2004; Mazaheri and Picton 2005), but no group differences. Greater phase consistency upon target presentation in the control group, relative to the clinical groups, may therefore suggest that controls consistently showed a reset and alignment in phase of theta oscillations (as indicated by the within-group increase in phase consistency from pre-target to post-target windows) over trials. This mechanism of phase-resetting has been previously associated with optimal behavioral performance (Biau et al. 2015; Lakatos et al. 2009; Palaniyappan et al. 2012). This process may be less consistent across trials in both disorders, as suggested by a lower degree of change from pre-target to post-target and lower phase consistency in theta oscillations in ADHD and BD groups than controls. These differences point to suboptimal regulation of this neural process in women with ADHD and women with BD.

**S-Table 1.** Descriptive statistics on study variables divided by group.

|  | **Baseline condition** | | | | | | **Fast-incentive condition** | | | | | |
| --- | --- | --- | --- | --- | --- | --- | --- | --- | --- | --- | --- | --- |
|  | **ADHD** | | **BD** | | **Ctrl** | | **ADHD** | | **BD** | | **Ctrl** | |
|  | **Mean** | **SD** | **Mean** | **SD** | **Mean** | **SD** | **Mean** | **SD** | **Mean** | **SD** | **Mean** | **SD** |
| **RTV** | 374.44 | 288.19 | 289.28 | 175.80 | 232.35 | 240.20 | 176.87 | 145.83 | 132.75 | 68.83 | 114.78 | 79.13 |
| **CNV** | -0.35 | 0.51 | -0.35 | 0.63 | -0.33 | 0.54 | -0.79 | 0.64 | -1.29 | 0.98 | -1.84 | 0.91 |
| **P3** | 531.58 | 459.56 | 501.53 | 359.17 | 531.72 | 496.91 | 465.10 | 307.51 | 427.19 | 310.80 | 618.51 | 388.27 |
| **Theta ERSP (0-500 ms)** | 1.22 | 0.69 | 1.41 | 0.83 | 1.54 | 0.92 | 0.74 | 0.76 | 1.02 | 0.80 | 1.31 | 0.98 |
| **Alpha ERSP (0-500 ms)** | -0.77 | 1.05 | -0.93 | 1.26 | -1.12 | 1.58 | -0.64 | 0.82 | -0.63 | 0.81 | -0.83 | 1.42 |
| **Alpha ERSP (500-1000 ms)** | -1.69 | 1.38 | -1.99 | 1.78 | -2.13 | 1.99 | -0.74 | 0.95 | -0.77 | 1.34 | -1.73 | 1.86 |
| **Beta ERSP (0-500 ms)** | -0.98 | 0.55 | -0.76 | 0.59 | -1.03 | 0.69 | -1.13 | 0.68 | -0.77 | 0.45 | -1.23 | 0.70 |
| **Beta ERSP (500-1000 ms)** | -1.27 | 0.71 | -0.94 | 1.41 | -0.94 | 0.92 | -0.59 | 0.71 | -0.52 | 1.13 | -0.58 | 1.02 |
| **Theta ITC (0-500 ms)** | 0.25 | 0.06 | 0.26 | 0.05 | 0.28 | 0.06 | 0.28 | 0.07 | 0.29 | 0.06 | 0.33 | 0.06 |

Abbreviations: ADHD, attention-deficit/hyperactivity disorder; BD, bipolar disorder; CNV, contingent negative variation; CP, centro-parietal region; Ctrl, control group; ERSP, event-related spectral perturbation; ITC, inter-trial phase coherence; MRT, mean reaction time; RTV, reaction time variability.SD, standard deviation of the mean.

**
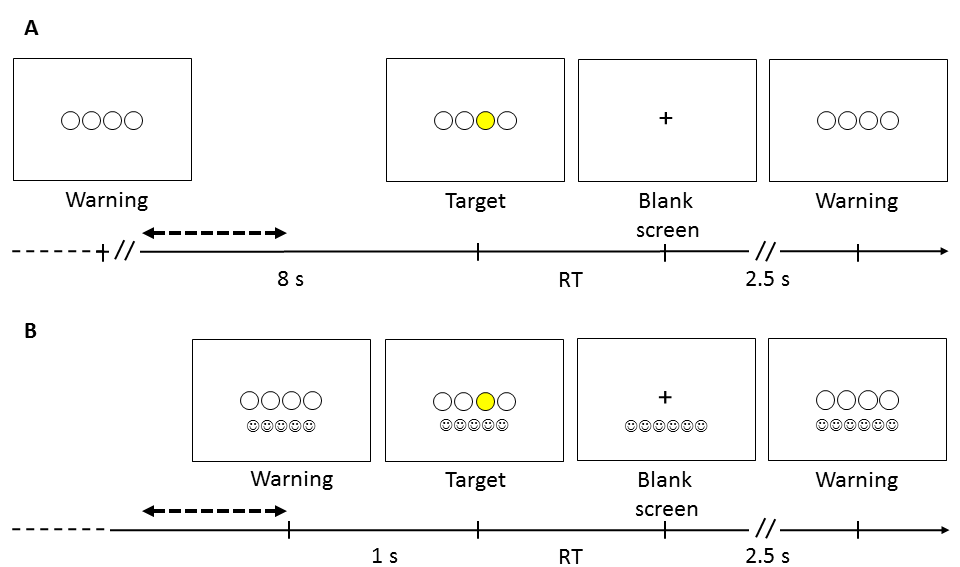
**

**Figure S1.** A schematic illustration of the temporal sequence of events in the (A) baseline and (B) fast‐incentive conditions of the Fast task.

Notes: In both conditions, the target remained on the screen up to 10 s until a response (response time [RT]). The double-headed dashed window corresponds to the pre-stimulus window used to normalize the event-related spectral perturbations (ERSPs).


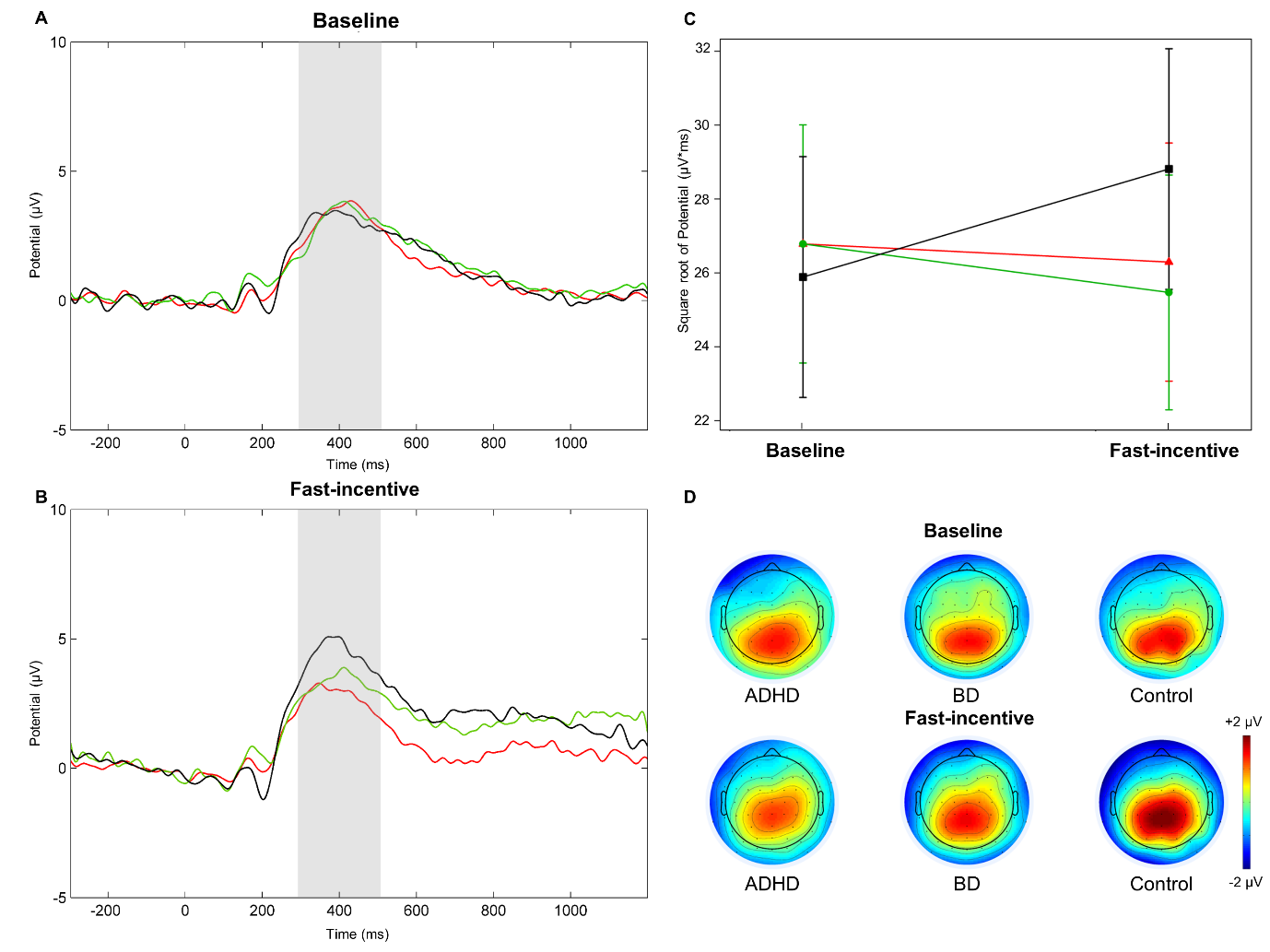
**Figure S2.** P3 amplitude measured at Pz in the 300–500 ms window in the ADHD (in red), BD (in green) and control (in black) groups across the baseline and fast incentive conditions of the Fast task. (A) Grand average in the baseline condition; (B) Grand average in the fast-incentive condition; (C) Condition effects by group; (D) Topographic maps by group at each condition.


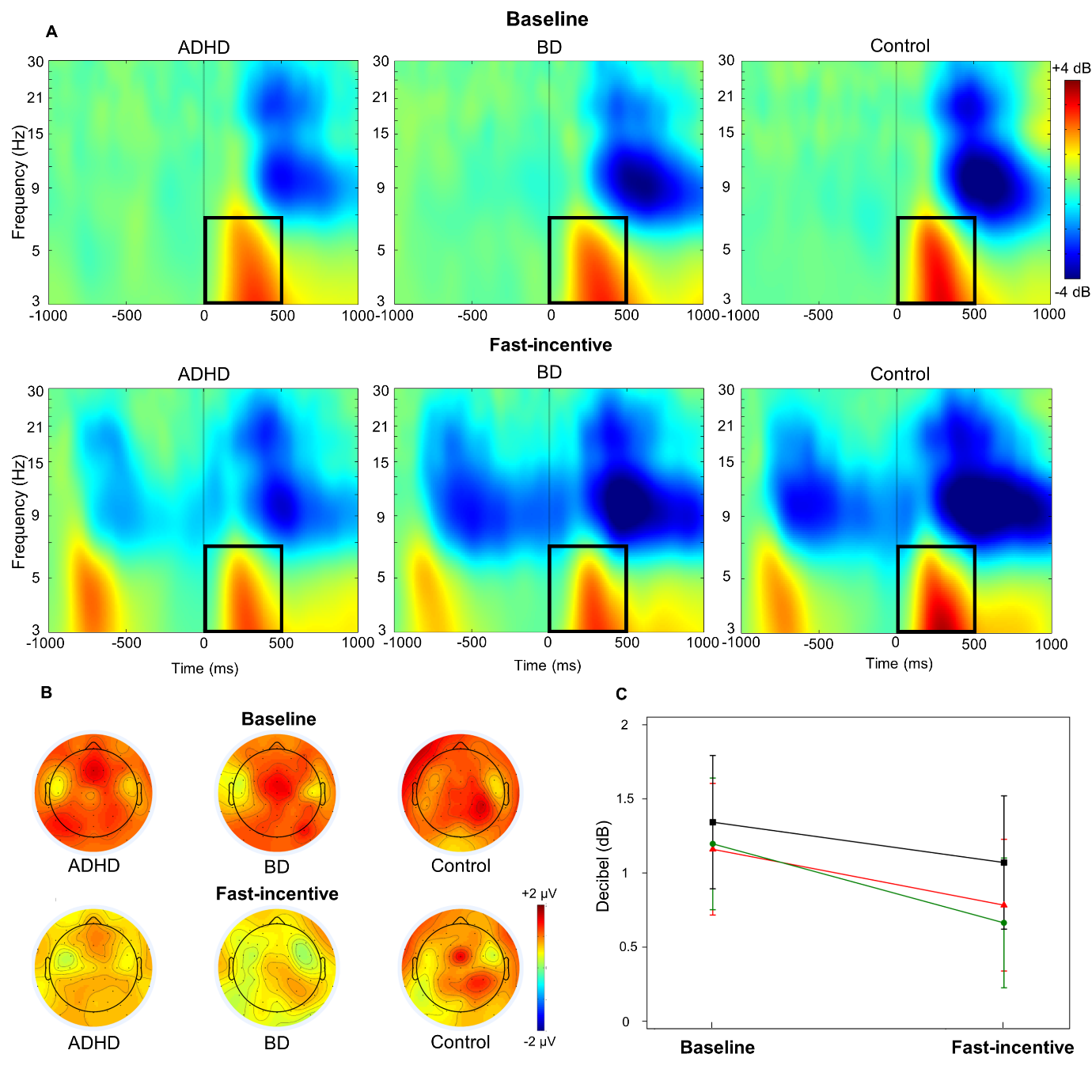
**Figure S3.** Theta event-related spectral perturbation (ERSP) at parietal regions by group across the baseline and fast incentive conditions of the Fast task. (A) ERSP in the baseline (top) and fast-incentive (bottom) conditions; (B) Topographic maps by group in the 0-500 ms window in each condition; (C) Condition effects in the 0-500 ms window in the ADHD (in red), BD (in green) and control (in black) groups.

**REFERENCES**

Andreou P et al. (2007) Reaction time performance in ADHD: improvement under fast-incentive condition and familial effects Psychol Med 37:1703-1715 doi:10.1017/s0033291707000815

Biau E, Torralba M, Fuentemilla L, de Diego Balaguer R, Soto-Faraco S (2015) Speaker's hand gestures modulate speech perception through phase resetting of ongoing neural oscillations. Cortex 68:76-85 doi:10.1016/j.cortex.2014.11.018

Cheung CH, McLoughlin G, Brandeis D, Banaschewski T, Asherson P, Kuntsi J (2017) Neurophysiological correlates of attentional fluctuation in attention-deficit/hyperactivity disorder. Brain Topogr. Epub ahead of print

Grandchamp R, Delorme A (2011) Single-trial normalization for event-related spectral decomposition reduces sensitivity to noisy trials. Front Psychol 2:236 doi:10.3389/fpsyg.2011.00236

Herrmann CS, Rach S, Vosskuhl J, Struber D (2014) Time-frequency analysis of event-related potentials: a brief tutorial. Brain Topogr 27:438-450 doi:10.1007/s10548-013-0327-5

Kuntsi J et al. (2013) Genetic analysis of reaction time variability: room for improvement?. Psychol Med 43:1323-1333 doi:10.1017/s0033291712002061

Lakatos P, O'Connell MN, Barczak A, Mills A, Javitt DC, Schroeder CE (2009) The leading sense: supramodal control of neurophysiological context by attention. Neuron 64:419-430 doi:10.1016/j.neuron.2009.10.014

Makeig S, Delorme A, Westerfield M, Jung TP, Townsend J, Courchesne E, Sejnowski TJ (2004) Electroencephalographic brain dynamics following manually responded visual targets. PLoS Biol 2:e176 doi:10.1371/journal.pbio.0020176

Mazaheri A, Picton TW (2005) EEG spectral dynamics during discrimination of auditory and visual targets. Brain Res Cogn Brain Res 24:81-96 doi:10.1016/j.cogbrainres.2004.12.013

Palaniyappan L, Doege K, Mallikarjun P, Liddle E, Francis-Liddle P (2012) Cortical thickness and oscillatory phase resetting: a proposed mechanism of salience network dysfunction in schizophrenia. Psychiatriki 23:117-129
